# The Black Necrotic Lesion Enhanced *Fusarium graminearum* Resistance in Wheat

**DOI:** 10.3389/fpls.2022.926621

**Published:** 2022-06-30

**Authors:** Lanfei Zhao, Peisen Su, Bingqian Hou, Hongyan Wu, Yanhui Fan, Wen Li, Jinxiao Zhao, Wenyang Ge, Shoushen Xu, Shiwen Wu, Xin Ma, Anfei Li, Guihua Bai, Hongwei Wang, Lingrang Kong

**Affiliations:** ^1^State Key Laboratory of Crop Biology, Shandong Key Laboratory of Crop Biology, College of Agronomy, Shandong Agricultural University, Tai’an, China; ^2^Department of Agronomy, Kansas State University, Manhattan, KS, United States; ^3^College of Agronomy, Liaocheng University, Liaocheng, China; ^4^Hard Winter Wheat Genetics Research Unit, USDA, Manhattan, KS, United States

**Keywords:** FHB, black necrotic lesions, metabolomics, transcriptomics, flavonoids, SA, MeJA

## Abstract

Fusarium head blight, mainly incited by *Fusarium graminearum*, is a devastating wheat disease worldwide. Diverse Fusarium head blight (FHB) resistant sources have been reported, but the resistance mechanisms of these sources remain to be investigated. FHB-resistant wheat germplasm often shows black necrotic lesions (BNLs) around the infection sites. To determine the relationship between BNL and FHB resistance, leaf tissue of a resistant wheat cultivar Sumai 3 was inoculated with four different *F. graminearum* isolates. Integrated metabolomic and transcriptomic analyses of the inoculated samples suggested that the phytohormone signaling, phenolamine, and flavonoid metabolic pathways played important roles in BNL formation that restricted *F. graminearum* extension. Exogenous application of flavonoid metabolites on wheat detached leaves revealed the possible contribution of flavonoids to BNL formation. Exogenous treatment of either salicylic acid (SA) or methyl jasmonate (MeJA) on wheat spikes significantly reduced the FHB severity. However, exogenous MeJA treatment prevented the BNL formation on the detached leaves of FHB-resistant wheat Sumai 3. SA signaling pathway influenced reactive oxygen species (ROS) burst to enhance BNL formation to reduce FHB severity. Three key genes in SA biosynthesis and signal transduction pathway, *TaICS1*, *TaNPR1*, and *TaNPR3*, positively regulated FHB resistance in wheat. A complex temporal interaction that contributed to wheat FHB resistance was detected between the SA and JA signaling pathways. Knowledge of BNLs extends our understanding of the molecular mechanisms of FHB resistance in wheat and will benefit the genetic improvement of wheat FHB resistance.

## Introduction

Plants coexist and coevolve with multiple pathogens. Since plants are immobile organisms and lack mammalian-like adaptive immunity, they have obtained various defense mechanisms including passive and active defense mechanisms during evolution to survive under adverse environments and retain their reproductivity (Abramovitch et al., 2006; Kiraly et al., 2007). The passive defense mechanisms take advantage of preexisting organization structures (Martin, 1964) and multiple chemical defense mechanisms to produce antimicrobial or toxic secondary metabolites, resistance-related proteins or peptides, and other antimicrobial compounds (Levin, 1976). The active defense mechanisms may include induction of oxidative burst (Lamb and Dixon, 1997), hypersensitive response (HR; Heath, 2000), and systemic acquired resistance (SAR; Ryals et al., 1996). Accumulation of phytoalexin-like compounds and metabolites can be triggered rapidly and directly in response to a pathogen attack (Thomma et al., 1999). The activation timing of the defense reactions and the strength of the defense responses usually play crucial roles in the development of resistance or susceptibility of a host plant (Kiraly et al., 2007; Ding et al., 2011).

Plant pathogens can be broadly divided into biotrophic and necrotrophic pathogens according to their lifestyles. Biotrophic pathogens gain nutrients from living host tissues; whereas necrotrophic pathogens kill host tissues to feed on the remains (Zhang et al., 2013). However, many pathogens are in between hemibiotrophic pathogens, and they can be either biotrophic or necrotrophic, depending on the conditions or the stages of their life cycles. *Fusarium graminearum* is a typical hemibiotrophic pathogen that causes wheat (*Triticum aestivum*) Fusarium head blight (FHB), a devastating wheat disease worldwide (Bai and Shaner, 1994). *F*. *graminearum* has a biotrophic phase at the early stages of FHB infection and a necrotrophic phase after the tissue death in the infection site (Bai and Shaner, 1994; Sorahinobar et al., 2016), which not only cause significant yield losses but also deteriorate grain quality. During infection, *Fusarium* can produce several types of trichothecene mycotoxins, such as deoxynivalenol (DON) and nivalenol (NIV), and these toxins are detrimental to humans and animals when the toxin-contaminated grains are used as foods and feeds (Bai and Shaner, 1994; Trail, 2009; Wang et al., 2020).

The molecular mechanisms underlying activation of plant defense responses to a hemibiotrophic pathogen are quite different from, even more complicated than, those for a biotrophic or necrotrophic pathogen. With the rapid development in “multi-omic” technologies, transcriptomics, proteomics and metabonomics have been used to elucidate mechanisms of FHB resistance (Wang et al., 2018; Su et al., 2020). Some studies showed that *F. graminearum* activated host defense genes in a similar manner to many other pathogens (Rudd et al., 2001; Kazan et al., 2012). Differentially expressed genes (DEGs) induced by *F. graminearum* infection mainly encode pathogenesis-related (PR) proteins, transporters, primary and secondary metabolisms, UDP-glycosyltransferases, lectins and regulators of oxidative burst, or compounds involved in hormone biosynthesis and signaling, phenylpropanoid biosynthesis, and other related pathways (Golkari et al., 2009; Kazan and Gardiner, 2018). Wang et al. (2018) conducted both transcriptome and hormone profiling and found that salicylic acid (SA) and jasmonic acid (JA) played positive roles in enhancing FHB resistance, and auxin and ABA were often associated with susceptibility; however, ethylene appeared to play dual roles during the *F. graminearum*-wheat interaction (Wang et al., 2018). More recently, Su et al. (2020) identified 789 differentially accumulated metabolites, including flavonoids, phenolamides, tryptamine derivatives, and phytohormones, and revealed altered expression of more than 100 known functional genes that are related to the biosynthesis or regulation in these pathways by analyzing both metabolomic and transcriptomic data (Su et al., 2020).

Although extensive studies have been conducted to reveal FHB resistance mechanisms, the conclusions on the *F. graminearum*-wheat interactions remain equivocal. In *tobacco mosaic virus* (TMV) disease, the local lesion has been considered one of the most notable resistance mechanisms in which the virus remains in a local infection and does not continue to spread to new cells after multiplying in several hundred cells around the entry point of the pathogen (Loebenstein, 2009). In many FHB-resistant lines, black necrotic lesions (BNLs) were observed around the entry sites of *F. graminearum*, with a few hyphae from the BNLs. In this study, we attempted to explore the possible relationship between wheat FHB resistance and BNLs using both metabolomic and transcriptomic approaches.

## Materials and Methods

### Plant Materials and Growth Conditions

The wheat cultivars Sumai 3, Jimai 22 and a set of wheat cultivars and breeding lines with different levels of FHB resistance (Ning 7840, Yangmai 158, Shengxuan 6, Haian 15-19, Am324925, Caizihuang, Guangtoumai, Chimianmai, Ningmai 6, Ningmai 9, Apogee73S2, Mimai, Tutoumai, Chimianmai, Huoshaomai, Zaoshiri, Laomangmai, Wugongmai, Huaiyang 05155, Jinmai 73, Linmai 7, Shannong 664, Yannong 19, Huaimai 33, and Taimai 198 and Apogee) were used in this study. Apogee (FHB susceptible NIL) and Apogee73S2 (FHB-resistant NIL) are two near-isogenic lines (NILs) contrasting in *Fhb1* alleles provided by Dr. David Garvin at USDA-ARS, Plant Science Research Unit, Saint Paul, MN, United States. All wheat seedlings were grown in a greenhouse at 20/25°C with a 16/8 h (light/dark) photoperiod. The wild-type *Nicotiana benthamiana* plants were grown in a growth chamber at 24/20°C with a 14/10 h day/night photoperiod.

### *Fusarium graminearum* Strains

The *F*. *graminearum* strain PH1-1 maintained in the State Key Laboratory of Crop Biology, Shandong Agricultural University was used as inoculum. The green-fluorescence-protein-labeled *F. graminearum* strain, eGFP-PH-1 obtained from Prof. Jinrong Xu of the Department of Botany and Plant Pathology at Purdue University, West Lafayette, IN, United States was used for tracking the pathogen spread. The *F*. *graminearum* isolates R40, R64, S52, and S66 were provided by Prof. Yuancun Liang at the Department of Plant Pathology, Shandong Agricultural University which were collected from 15 cities in Shandong Province. The four isolates were selected from the 93 *F*. *graminearum* based on their virulence to Sumai 3. S52 and S66 were highly virulent, and R40 and R64 were low virulent to Sumai 3.

### Evaluation of *F. graminearum* Resistance in Detached Spikes and Leaves

The wheat spikes were inoculated by single-floret inoculation as described by Bai and Shaner (1994) with minor modifications (Kong et al., 2007). Briefly, one basal floret of the middle spike was inoculated with 10 μl of an *F*. *graminearum* conidia suspension (5–10 × 10^4^ conidia mL^–1^) at anthesis, and then sealed with transparent plastic bags to maintain moisture for 48 h. The number of diseased spikelets and rachises was recorded at 21 DAI.

A detached leaf assay described by Browne and Cooke (2004) was used to screen FHB resistance in wheat with minor modifications. Briefly, wheat seedlings were grown in a growth chamber at 22 and 19°C day/night with 12 h photoperiod and 45–50% relative humidity. A 4.5 cm segment from the mid-section of the first leaf was collected at the two-leaf stage. Each detached leaf segment was injured by punching the center of the leaf segment using a pipette tip and arched in the top of a well in a 96-well plate. The plate was then moved gently to a plastic tray (30 cm × 50 cm × 15 cm) filled with water that submerged both ends of the leaf segment in the water. The tip injured site on the leaf segment was added with 3 μl of a *Fusarium* conidia suspension at 5–10 × 10^4^ conidia mL^–1^. Then, the tray was covered with a transparent plastic wrap to maintain moisture at 25–28°C in a growth chamber for 3 days before the length of saprophytic spots was measured.

### Exogenous Hormone Treatment on Detached Spikes

The consistent spikes of FHB susceptible cultivar Jimai 22 were selected at anthesis and cut from the base of the stem. The spikes were treated in a solution containing 2 mM SA or 100 μM MeJA at 0, 12, 24, and 48 HAI of *F*. *graminearum* PH1-1 using a single floret inoculation. The spikes were covered with thin plastic wrap and then maintained in a 25°C incubator for 2–3 days. Water was used as the negative control. The number of FIR that directly connected to the inoculated spikelet was counted at 4 DAI and DR was recorded at 7 DAI. PSS of the detached spikes was counted at 15 DAI. At least 20 spikes per replication were counted with three replicates in each treatment. Student’s *t*-test analysis was used to compare the differences among exogenous hormone treatments, and a significant difference was claimed at *p* < 0.05.

### Preparation of Scanning Electron Microscopy

The tissue for scanning electron microscopy was prepared as described by Kang and Buchenauer (2000), with minor modifications. The inoculated rachises of Sumai 3 and Jimai 22 were sampled at 12 DAI with PH1-1. The samples were fixed with 4% (v/v) glutaraldehyde in 50 mM phosphate buffer (PH = 6.8) for 24 h and rinsed for five cycles with phosphate buffer (PH = 6.8) at 20 min per cycle. Subsequently, samples were postfixed in 1% (w/v) osmium tetroxide in 50 mM phosphate buffer (PH = 6.8) overnight at 4°C and rinsed for five cycles with phosphate buffer (pH 6.8) at 20 min per cycle, then rinsed with a graded of ethanol and isopentyl acetate series. After dehydration, the sample was dried, mounted on a stub, sputter coated with gold-palladium, and viewed using a Zeiss 100 scanning electron microscope (JEOL, JSM-6610 LV) operating at 15 kV. Each treatment had three replications.

### Transcriptional Profiling

The detached leave segments described previously were used for transcriptional profiling. The first leaves of FHB-resistant wheat cultivar Sumai 3 were inoculated with conidia suspensions of four *F. graminearum* isolates R40, R64, S52, or S66 with different virulence and harvested at 4 DAI. Total RNA samples were isolated using TRIzol reagent (Invitrogen Corporation, Carlsbad, CA, United States) and RNA sequencing was done in the Berry Genomics Corporation Company (Beijing, China) using an Illumina HiSeq™ 2000 sequencer. The sequence reads were aligned to the Chinese Spring reference genome v1.0 (International Wheat Genome Sequencing Consortium [IWGSC], 2018). The differentially expressed genes (DEGs) were analyzed by comparing each treated sample with its mock-inoculated control using the DEGseq R package (Mortazavi et al., 2008). The genes were considered to show a statistically significant difference based on a false discovery rate (FDR) < 0.05. The raw expression values were converted into log2 ratios.

MapMan was specifically designed to cover plant-specific pathways and processes^1^. MapMan pathway analyses were performed using the Log_2_ fold change of common DEGs induced by both R40 and R64 (RR) or both S52 and S66 (SS) to determine BNL-related pathways. Custom specific mapping file for the MapMan based on the wheat sequencing output was created using the Mercator pipeline (Lohse et al., 2014)^2^. MapMan v3.5.1R2 (Thimm et al., 2004) was used to visualize expression changes of DEGs to multiple MapMan functional categories.

### Metabolomic Analysis

The detached leaf samples were inoculated with an *F*. *graminearum* conidia suspension as described previously. Leaf tissue was collected including the saprophytic spot and 2–3 mm adjacent leaf tissue at 4 DAI. The samples were prepared and extracted as previously described (Chen et al., 2013) and analyzed using an LC-ESI-MS/MS system (HPLC, Shim-pack UFLC SHIMADZU CBM20A system^3^; MS, Applied Biosystems 4000 Q TRAP^4^). The analytical conditions were as follows: HPLC: column, shim-pack VP-ODS C18 (pore size 5 μm, length 2 mm × 150 mm); solvent system, water (0.04% acetic acid): acetonitrile (0.04% acetic acid); gradient program, 100:0 V/V at 0 min, 5:95 V/V at 20 min, 5:95 V/V at 22 min, 95:5 V/V at 22.1 min, 95:5 V/V at 28.0 min; flow rate, 0.25 ml min^–1^; temperature, 40°C; and injection volume: 5 μl. The effluent was connected to an ESI-triple quadrupole-linear ion trap (Q TRAP)-MS. Linear Ion Trap (LIT) and triple quadrupole (QQQ) scans were acquired on a triple quadrupole-linear ion trap mass spectrometer (Q TRAP), API 6500 Q TRAP LC/MS/MS System, equipped with an ESI Turbo Ion-Spray interface, operating in a positive ion mode and controlled by Analyst 1.6 software (AB Sciex, Framingham, MA, United States).

Metabolite data were log_2_-transformed before statistical analysis to improve normality. To identify the differentially accumulated metabolites induced by different virulent *F. graminearum* isolates, *F. graminearum*-infected samples were compared with the mock-inoculated control (CK). The principal component analysis (PCA) was carried out by SPSS for Windows (Version 25, SPSS Inc., Chicago, IL, United States). Differences in the metabolites between wheat leaves from treated and control samples were determined using Welch’s *t*-test (*p* < 0.01) in ZS97 and IRAT10 (Chen et al., 2013). The metabolites were identified as significantly enriched or depleted in content and were set with thresholds of variable importance in fold-change ≥1.5 or ≤0.5.

### Endogenous Salicylic Acid and Jasmonic Acid Content Measurements

To measure endogenous SA and JA content, a central spikelet in a spike of Sumai 3 at anthesis was inoculated with *F. graminearum* PH1-1 using single-floret inoculation (Bai and Shaner, 1994; Kong et al., 2007). The inoculated spikelets were sampled at 0, 12, 24, 48, 72, and 96 HAI to extract and quantify SA and JA using an HPLC-ESI-MS/MS separation (Agilent 1200, Agilent Technologies, Palo Alto, CA, United States) as described by Ding et al. (2011). The standard curves for SA and JA quantification were generated using a series of SA and JA (Sigma, St. Louis, MO, United States) dilutions. All experiments were performed with two biological replicates and three technical replicates.

### Identification of Reactive Oxygen Species in Detached Leaves

The ROS levels were measured using the cell-permeable fluorescent dye DCFH-DA (2′,7-dichlorofluorescein diacetate) as described by Tiwari and Kakkar (2009) with minor modification. Briefly, detached leaves were incubated in 5 μM DCFH-DA for 45 min using a pH 7.5 phosphate buffer in the dark at 3 DAI of *F*. *graminearum*. The samples were rinsed with 70% ethanol and photographed using a BX60 light microscope (Olympus, Japan).

### Exogenous Treatments of Metabolites and Hormones on the Detached Leaves

To study the function of metabolites and hormones in FHB resistance, exogenous metabolites and hormones were applied on detached wheat leaves of Jimai 22, Kenong 199, Huaimai 33, and Taimai 198, respectively. The detached leaves were placed separately in a solution containing 2 mM SA, 100 μM MeJA, 50 mM apigenin, neohesperidin, catechin, proanthocyanidins, or spermidine. Water treatment was used as a control. The treated and controlled detached leaves were prepared and inoculated with *F*. *graminearum* PH1-1 as described previously, then placed in a plastic tray (30 cm × 50 cm × 15 cm) and sealed with transparent plastic wrap to maintain high moisture content for 3 days in a growth chamber set at 25°C. The length of necrotic spots was measured in 20 leaf segments per replicate for three replicates per treatment.

### Gene Expression Analysis

The two NILs, Apogee and Apogee73S2, were inoculated with *F*. *graminearum* PH1-1 (5–10 × 10^4^ conidia mL^–1^) as previously described, and the inoculated spikes were harvested at 0, 12, 24, 48, and 72 HAI to isolate total RNA using TRIzol reagent (TransGen, Beijing, China). The total RNA was then reverse-transcribed into cDNA using a TransScript^®^ One-Step gDNA Removal and cDNA Synthesis Kit (TransGen, Beijing, China) and oligo(dT) primers following the manufacturer’s instructions. The expression of targeted genes was profiled using qRT-PCR in a Roche LightCycler^®^ 480 (Roche, Mannheim, Germany). The housekeeping gene β*-Actin* was used as an internal reference. The threshold cycle (CT) values were used to calculate the fold-changes of relative expression accumulation using the formula 2^–ΔΔ^*^CT^* and standard errors. Each treatment had three biological replicates, and each biological replicate had three technical replicates to reduce the error. All primers used in this study were listed in Supplementary Table 1. Student’s *t-test* analysis was used to compare the differences in the gene expression levels among different time points.

### Functional Evaluation of Key Genes in Salicylic Acid Pathway Using *Barley Stripe Mosaic Virus*-Induced Gene Silencing

Transient silencing of key genes in the SA pathway was carried out using the *barley stripe mosaic virus-induced* gene silencing system (Yuan et al., 2011; Fan et al., 2019). Briefly, the specific short fragments of *TaICS1*, *TaPAL2*, *TaNPR1*, *TaNPR2*, and *TaNPR3* were obtained from the cDNA of Sumai 3 using corresponding primer pairs, respectively (Supplementary Table 3). The fragments were cloned into the vector pCa-γbLIC (pCa-γb:target-gene) and transformed into *Agrobacterium EHA105* using the previously described method (Yuan et al., 2011). The *Agrobacterium* mixtures containing equal amounts of pCaBS-α, pCaBS-β, and pCa-γb-target-gene were used to inoculate *N. benthamiana* at the four-leaf stage to generate the recombinant virus initially. At 7–10 DAI, the tobacco leaves with mosaic spots were harvested and ground in the pH 7.2 phosphate buffer saline (PBS) containing 1% celite. The leaf sap was mechanically rub-inoculated onto the flag leaves of Sumai 3 (Fan et al., 2019). The transcript abundances of target genes were detected by semi-quantitative RT-PCR, and 18S rRNA was used as an internal control. Each treatment had three biological replicates, and each biological replicate had three technical replicates. The number of scabbed spikelets was recorded from 7 to 30 DAI with *F. graminearum*. Each treatment had three replicates with 10 spikes per replicate.

## Results

### Black Necrotic Lesion Restricted *F. graminearum* Spread in Wheat

**FIGURE 1:**
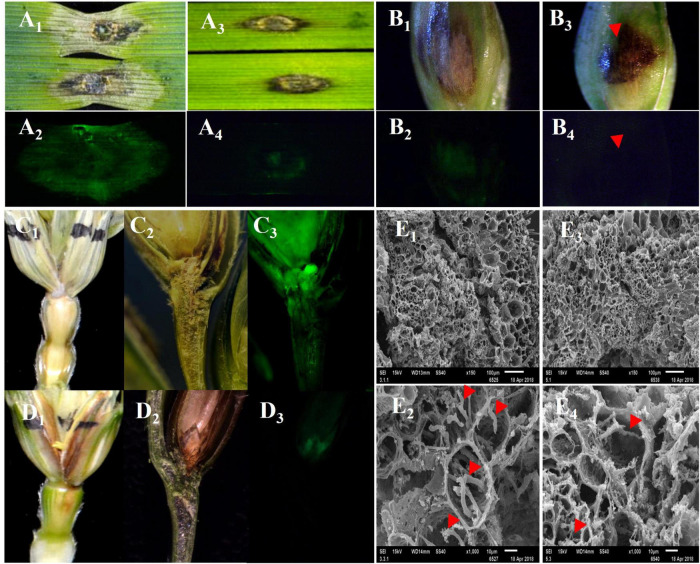
The disease symptoms and movement of *F. graminearum* in detached leaves, glumes, and spikes of susceptible (Jimai 22) and resistant (Sumai 3) plants after infection of *F. graminearum* eGFP-PH-1. The black necrotic lesions (BNLs) were detected in Sumai 3, which effectively limited the size of disease symptoms and hypha spread of *F. graminearum* among tissues. **(A_1_,A_2_,B_1_,B_2_,C_1_–C_3_)** Show detached leaves, glume, and spike of Jimai 22 after infection of *F. graminearum* that were photographed under regular light and green fluorescent light, respectively. **(A_3_,A_4_,B_3_,B_4_,D_1_–D_3_)** Show the detached leaves, glume, and spikes of Sumai 3 after inoculation of *F*. *graminearum* that were photographed under regular light and green fluorescent light, respectively. **(E_1_–E_4_)** Show the organizational structure of the nodes which connect the inoculated floret to the rachis of Jimai 22 and Sumai 3, respectively, at 12 DAI under SEM. The red arrows point to hyphae of *F. graminearum* in the rachis.

The FHB symptoms on the leaves, glumes, and spikes were significantly different between the FHB-resistant wheat cultivar “Sumai 3” and the susceptible wheat cultivar “Jimai 22” after they were inoculated with different isolates of *F. graminearum*. In Jimai 22, a large water-soaked spot was observed on a detached leaf 4 days after inoculation (DAI), and a dry, chalky spot appeared on the glume at 7 DAI and spread to rachis at 12 DAI (Figures 1A_1_–C_1_). In Sumai 3, however, BNLs appeared on a detached leaf, glume, and rachis (Figures 1A_3_,B_3_,D_1_). When these tissues were inoculated with the green fluorescence-labeled *F. graminearum* strain eGFP-PH-1, a large mass of hyphae was observed around the water-soaked spots on the leave, glum and spike of Jimai 22 (Figures 1A_2_,B_2_,C_3_), but very few hyphae were observed in BNLs on these tissues of Sumai 3 (Figures 1A_4_,B_4_,D_3_). In the longitudinal section of rachis and floret of Jimai 22, hyphae passed the node between floret and rachis and quickly spread to the adjacent rachises (Figures 1C_2_,C_3_); whereas the hyphae mainly appeared on the surface of the inoculated floret and rarely spread to rachis in Sumai 3 (Figures 1D_2_,D_3_). Under scanning electron microscope (SEM), much more hyphae grew in the infected rachis of Jimai 22 than in Sumai 3, and hyphae in the rachis of Sumai 3 were thinner and weaker than in Jimai 22 (Figures 1E_2_,E_4_). These results support that the black substances in BNLs may slow down or inhibit the spread of *F. graminearum* among different wheat tissues.

To investigate the relationship between BNLs and the spread of *F*. *graminearum* in different wheat tissues, 19 moderately to highly FHB resistant and 8 highly susceptible wheat cultivars were phenotyped for FHB symptoms on spikes at 21 DAI. All the 19 FHB-resistant cultivars showed a lower percentage of symptomatic spikelets in a spike (PSS), ranging from 6.4 to 45.4%, than these susceptible cultivars, ranging from 54.5 to 89.3%, and had obvious BNLs in the inoculated spikelet and sometimes in the adjacent rachis of most FHB-resistant cultivars. Sumai 3 and Ning 7840, with the highest FHB resistance among tested cultivars, showed BNLs on the inoculated spikelets as early as 3 DAI. Only Wugongmai, a moderately resistant cultivar, did not show BNLs even at 21 DAI. All eight susceptible wheat cultivars showed high PSS and chalky-dry symptoms on rachises, except Kenong 199 which showed some black lesions in the inoculated spikelets and neighboring rachises at 10 DAI (Supplementary Figure 1). That *F*. *graminearum* infection-induced BNLs in most FHB-resistant cultivars but not in most susceptible cultivars suggests that BNL may play an important role in wheat resistance to FHB.

### Differentially Expressed Genes and Accumulated Substances Induced by *F*. *graminearum* Isolates With Different Degrees of Virulence

**FIGURE 2:**
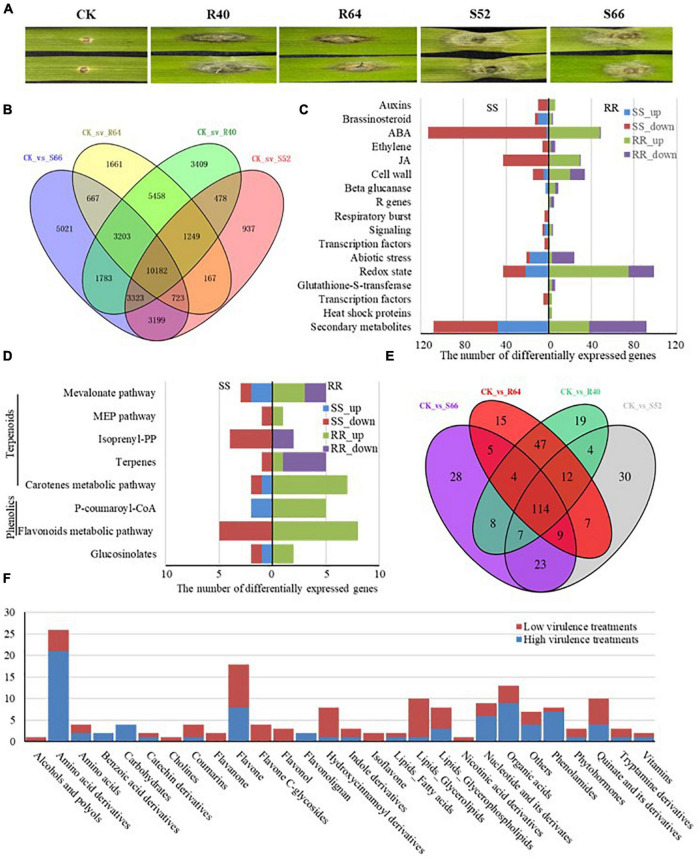
Overview of significantly differentially expressed genes (DEGs) and the accumulated metabolites in detached leaves of Sumai 3 treated with the *F*. *graminearum* isolates R40, R64, S52, and S66. **(A)** The symptoms on the detached leaves inoculated with H_2_O, low virulent isolates (R40 and R64), and high virulent isolates (S52 and S66). **(B)** Venn diagram of significantly DEGs induced by R40, R64, S52, and S66 compared with CK. **(C)** A number of significant DEGs were mapped to the corresponding pathways in biotic stress pathways. **(D)** A number of significant DEGs were mapped to the secondary metabolism pathway. **(E)** A Venn diagram of the different metabolites induced by R40, R64, S52, and S66 compared with CK. **(F)** Number of specific metabolites in different categories after inoculating with high- and low-virulence *F*. *graminearum*.

Significantly different symptoms were observed on the detached leaves of Sumai 3 at 4 DAI with the four *F*. *graminearum* isolates (R40, R64, S52, S66). The mock-inoculated leaves (CK) did not show any symptoms and the isolates R40 and R64 induced small BNLs around the inoculation points on the leaves. However, the isolates S52 and S66 induced large water-soaked spots surrounding inoculation points, therefore, were more virulent than R40 and R64 in Sumai 3 (Figure 2A).

To identify DEGs and related pathways induced by *F*. *graminearum*, comparisons of transcript profiles between the *F*. *graminearum* isolates S52, S66, R40, and R64 inoculated and mock-inoculated Sumai 3 leaf tissues identified 41,460 unique DEGs, with 20,258 between S52 and CK, 28,101 between S66 and CK, 29,085 between R40 and CK and 23,310 between R64 and CK. Among these, 10,182 genes were differentially expressed in all four isolates, 5,458 genes were differentially expressed in the R40 and R64 (low virulence) inoculated samples (Supplementary Table 2), 3,199 genes were differentially expressed in the S52 and S66 (high virulence) inoculated samples (Supplementary Table 3), and 11,019 DEGs were uniquely induced by a single isolate including 937 genes induced by S52, 5,021 genes by S66, 3,409 genes by R40 and 1,661 genes by R64 (Figure 2B). Among these DEGs, 375 genes induced by both R40 and R64 and 396 by both S52 and S66 were related to the biotic stress pathways. The upregulated DEGs by the two low virulent isolates (R40 and R64) mainly code proteins for phytohormone signaling pathways including auxin, brassinosteroids, abscisic acid (ABA), ethylene (ET), and jasmonic acids (JA) pathways (Figure 2C) and some proteins for the redox state, secondary metabolites, cell wall, and abiotic stress pathways in responses to *F*. *graminearum* infection; however, most S52 and S66 induced DEGs were downregulated in the ET, ABA, and JA pathways (Figure 2C), suggesting that phytohormone signaling pathways played an important role in the interaction between *F*. *graminearum* and wheat.

In Sumai 3, 20 DEGs induced by S52 and S66 and 35 DEGs induced by R40 and R64 were mapped in the secondary metabolism pathways, in particular flavonoids metabolic pathway in which all the five upregulated DEGs (Traescs2D01G530600, Traescs2B01G048400, Traescs2A01G468200, Traescs6B01G056900, Traescs6D01G048400) were induced by R40 and R64, and all the eight downregulated DEGs (Traescs3A01G253800, Traescs3D01G254700, Traescs5A01G475600, Traescs7B01G310900, Traescs7A01G411700, Traescs2D01G512700, Traescs2A01G511300, Traescs7A01G212800) were induced by S52 and S66 (Figure 2D). The results suggest that the flavonoids metabolic pathway is differentially regulated between the wheat-low virulent *F*. *graminearum* interaction system and the wheat-high virulent *F*. *graminearum* interaction system, which may contribute to the regulation of BNL formation in responses to *F*. *graminearum* infection.

Metabolomic analyses on the detached leaf tissues of Sumai 3 that were collected 4 DAI by the isolates R40, R64, S52, or S66 identified 789 metabolites with known structures. A total of 206, 198, 215, and 213 metabolites were significantly differentially accumulated in S52, S66, R40, and R64 inoculated samples, respectively (Su et al., 2020). Compared with the mock-inoculated control (CK), 30, 28, 19, and 15 metabolites were specifically accumulated in S52, S66, R40, and R64 inoculated samples, respectively; 23 metabolites were accumulated in both S52 and S66 inoculated samples; 47 metabolites were accumulated in both R40 and R64 inoculated samples; 114 were common metabolites accumulated in all the samples that were inoculated with the four different isolates (Figure 2E).

In the R40 and R64 (RR cluster) treated samples and S52 and S66 (SS cluster) treated samples, 81 species of *Fusarium*-induced metabolites were identified and further functionally classified, respectively (Supplementary Tables 4, 5). Based on the structural features of each metabolite, the RR cluster contained 21 categories of metabolites and the SS cluster had 25 categories. Many of the metabolites were amino acid derivatives, flavones, and quinate derivatives, suggesting that both highly and lowly virulent *F*. *graminearum* isolates induced the accumulation of these metabolites. Since highly virulent isolates did not produce BNL, these metabolites may not be the causal agents for BNL.

In comparison of the metabolite categories between RR and SS clusters, two species of benzoic acid derivatives, four species of carbohydrates, and two species of flavonolignans were identified in the samples only from the RR cluster. In addition, seven species of phenolamides were identified in the samples from the RR cluster with only one of these species from the SS cluster. These results suggest that these metabolites were induced specifically by infection of the high virulent *F*. *graminearum*, which might result in water-soak spots on wheat. However, one species each of alcohols and polyols, two species of flavanones, four species of flavone C-glycosides, three species of flavonols, two species of isoflavones, and one species of nicotinic acid and its derivatives were identified from low-virulent-isolate-inoculated samples (Figure 2F), suggesting that flavonoids and its derivatives might contribute significantly to BNL formation in FHB-resistant plants.

### Responses of Exogenous Metabolite Treated Wheat Detached Leaves to *F*. *graminearum* Infection

**FIGURE 3:**
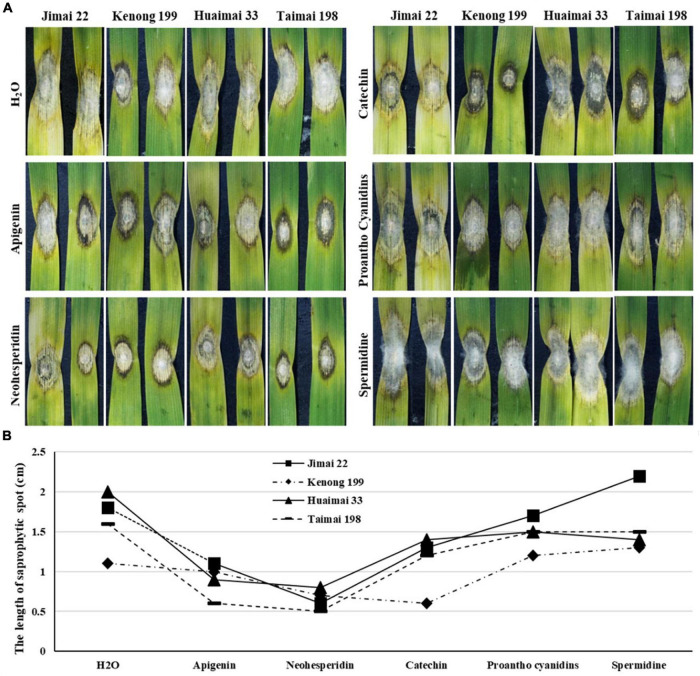
Response to *F. graminearum* PH1-1 infection to exogenous metabolite treatments on the detached leaves of four different wheat cultivars (Jimai 22, Kenong 199, Huaimai 33, and Taimai 198) at 4 DAI. **(A)** Detached wheat leaves were treated with H_2_O, 50 μM of apigenin, neohesperidin, catechin, proanthocyanidins, and spermidine after *F*. *graminearum* inoculation, respectively. **(B)** Length of the saprophytic spots on the detached leaves of Jimai 22, Kenong 199, Huaimai 33, and Taimai 198 at 4 DAI treated with H_2_O and 50 μM of apigenin, neohesperidin, catechin, proanthocyanidins, and spermidine, respectively. Each replicate comprised at least 20 detached leaves.

The leaves were detached from four highly FHB susceptible wheat cultivars (Jimai 22, Kenong 199, Huaimai 33, and Taimai 198) and treated with 50 μM each of apigenin, neohesperidin, catechin, proanthocyanidin, and spermidine, individually. Among these chemicals, apigenin and neohesperidin belong to flavonoids, proanthocyanidin belongs to anthocyanins, catechin belongs to catechin derivatives, and spermidine belongs to phenolamides. All the chemical-treated leaves were then inoculated with the *F*. *graminearum* PH1-1. Although variation in sizes of BNLs was observed among cultivars within a treatment, obvious variation was observed among treatments. The catechin treatment limited *F*. *graminearum* spread in detached leaves of all wheat cultivars with the smallest BNLs in Kenong 199. The apigenin and neohesperidin-treated leaves showed moderate BNLs around the *F*. *graminearum* inoculation sites but had significantly shorter saprophytic spots than negative H_2_O control on all four inoculated wheat cultivars at 4 DAI (Figure 3). Proanthocyanidins-treated leaves showed a similar phenotype to H_2_O treated CK in all cultivars. The spermidine-treated samples showed excessive hypha growth on the saprophytic spots and longer saprophytic spots than H_2_O-treated CK in all four cultivars. These results indicated that exogenous application of flavonoids may induce BNLs to restrain fungal growth on wheat leaves.

### The Relationship of Salicylic Acid and Jasmonic Acid to Wheat Fusarium Head Blight Resistance

In the susceptible wheat Kenong 199, the mean length of saprophytic spot (0.73 cm) in the H_2_O-treated detached leaves was longer than in the SA-treated samples (0.57 cm), but was shorter than methyl jasmonate (MeJA)-treated leaves (0.95 cm) at 4 DAI of *F. graminearum* PH1-1 with obvious BNLs only in the SA-treated samples (Supplementary Figures 2A,B). In the resistant cultivar Sumai 3, small BNLs (0.45 and 0.46 cm) were observed in H_2_O- and SA-treated leaves, respectively; whereas the MeJA-treated leaves showed serious etiolation with the longest saprophytic spots (1.53 cm) among the six treatment combinations (Supplementary Figures 2A,C). These results indicated that exogenous application of SA induced BNLs to restrain fungal growth on wheat leaves; in contrast, JA facilitated the antagonistic interactions between the SA and JA signaling pathways to promote *F. graminearum* growth. In addition, the small BNLs in Sumai 3 showed very strong ROS signals around the edge of the BNL at 4 DAI (Supplementary Figure 3A_2_), but Jimai 22 had relatively large saprophytic spots with very weak ROS signals in the spot (Supplementary Figure 3B_2_), suggesting that ROS burst might contribute to the BNL formation to limit the growth of *F. graminearum*.

**FIGURE 4:**
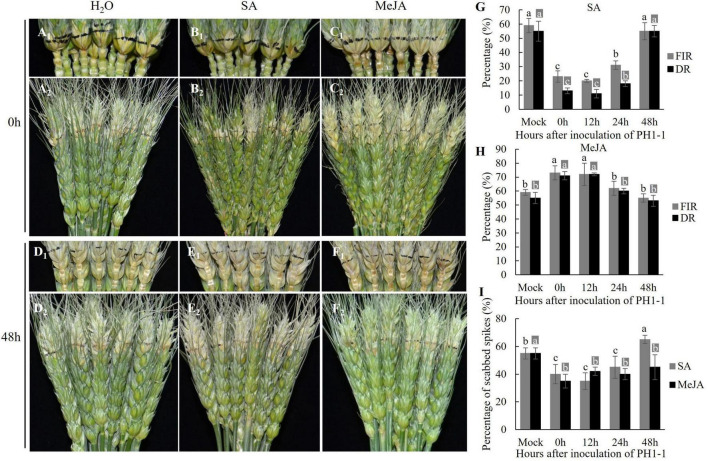
The comparisons of FHB symptoms, first infected rachises (FIR), and dead rachises (DR) on the spikes of Jimai 22 that were applied with exogenous SA and MeJA at 0–48 h after inoculation (HAI) with PH1-1. **(A_1_–F_1_)** Showed the infected rachises of the inoculated spikes at 7 days after inoculation (DAI) and **(A_2_–F_2_)** showed the infected spikelets in inoculated spikes at 7 DAI. All these samples were treated with H_2_O (Mock), SA, and MeJA, respectively. **(G,H)** Percentage of FIR and DR in the infected spikes of Jimai 22 that were applied with H_2_O, SA, and MeJA at 0 h, 12 h, 24 h, and 48 h after PH1-1 inoculation at 4 DAI and 7 DAI, respectively. **(I)** PSS of infected spikes of Jimai 22 at 15 DAI that was applied with H_2_O, SA, and MeJA at 0, 12, 24, and 48 HAI of PH1-1. Error bar shows standard deviation. In **(G–I)**, different letters on the top of each bar in the same color (dark or light) indicate significant differences at *p* = 0.05 (LSD) among mean FIR, DR, or PSS at inoculation time points, respectively.

Application of exogenous SA at 0, 12, and 24 HAI with PH1-1 restrained the spread of the pathogen from the inoculated spikelet to the adjacent rachis on the detached spikes of Jimai 22 (Figure 4). When SA was applied at 0, 12, and 24 HAI, the percentage of the first infected rachis (FIR) was significantly lower (23, 20, and 31%, respectively) than H_2_O control (59%) at 4 DAI with PH1-1; however, FIR was similar (55%) to H_2_O control when SA was applied at 48 HAI with PH1-1 (Figure 4G). The percentage of dead rachises (DR) followed the same pattern as FIR. Also, the percentage of scabbed spikes (PSS) in these SA-treated samples at 0, 12, and 24 HAI with PH1-1 were significantly lower than the H_2_O control. However, the SA-treated spikes at 48 HAI with PH1-1 produced higher PSS (65%) than the H_2_O control (55%) (Figure 4I).

In the MeJA-treated samples, the MeJA slightly aggravated the spread of *F*. *graminearum* in the spikes that were treated with MeJA at 0, 12, and 24 HAI with PH1-1. However, both FIR and DR of the spikes that were treated with MeJA at 48 HAI were the same as H_2_O control (Figure 4H). Most MeJA-treated spikes (0, 24, and 48 HAI) had lower PSS than those treated with SA with the lowest PSS for 0 HAI treatment (Figure 4I). Therefore, early exogenous application (0–24 HAI) of SA significantly enhanced wheat FHB resistance, while exogenous application of MeJA could effectively slow down the fungal spread within a spike after the initial disease spread to rachises if it is applied within 48 h of FHB infection.

To study the relationship between *F*. *graminearum* infection and SA or JA content accumulated in the resistant plants, endogenous SA and JA contents were measured in dry spike tissues of Sumai 3 at 0, 12, 24, 48, 72, and 96 after inoculation with *F. graminearum* PH1-1. SA content started with 73.8 ng/mg at 0 HAI, quickly increased to the peak (125.1 ng/mg) at 12 HAI, then quickly dropped to 22.3 ng/mg at 48 HAI (Supplementary Figure 4); in contrast, JA content in Sumai 3 started (0 HAI) at low levels of 3–3.9 ng/mg, decreased slowly at 24 HAI, then jumped 12.1 ng/mg at 24 HAI, and reached 15.8 ng/mg at 96 HAI. These data suggested an antagonistic interaction between the SA and JA signaling pathways, and that high SA content in the early stage of the fungal infection is likely associated with FHB resistance.

### Expression Patterns of Key Genes for Salicylic Acid Synthesis and Signaling

**FIGURE 5:**
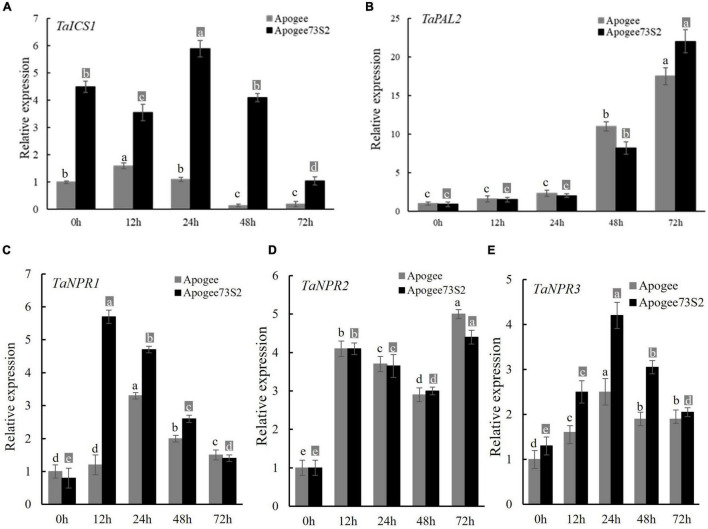
Expression of *TaICS1* **(A)** and *TaPAL2* **(B)** for SA synthesis, and *TaNPR1* **(C)**, *TaNPR2* **(D)**, and *TaNPR3* **(E)** for SA signaling transduction in the spike of the Apogee (Fhb1 susceptible NIL) and Apogee73S2 (Fhb1 resistant NIL) at 0, 12, 24, 48, and 72 h after inoculation with *F. graminearum* PH1-1. Error bar shows standard deviation. Different letters on the top of each bar in the same color (dark or light) indicate significant differences at *p* = 0.05 (LSD) among relative expressions at inoculation time points.

The expression patterns of genes encoding an isochorismate synthase (*TaICS1*) and a phenylalanine ammonia-lyase (*TaPAL2*) for SA synthesis and *TaNPR1*, *TaNPR2*, and *TaNPR3* for signaling transduction were analyzed using the tissues from two *Fhb1*-near-isogenic lines (NILs), Apogee (*Fhb1*^–^) and Apogee73S2 (*Fhb1*^+^), collected at 0, 12, 24, 48 and 72 HAI with PH1-1. *TaICS1* expression in the resistant NIL Apogee73S2 was significantly higher than in the susceptible NIL Apogee at all the five-time points, with the peak at 24 HAI (Figure 5A). Had non-differential expression of *TaPAL2* between the NILs (Figure 5B) suggests *TaPAL2* may not associate with FHB resistance. Therefore, that higher endogenous SA content coincided with the higher expression of *TaICS1* in the FHB-resistant NIL suggests that the *TaICS1* gene is likely a key gene in the SA synthesis pathway and it may play an important role in wheat FHB resistance.

Among three *TaNPR* genes, *TaNPR1* showed significant differential expression between the two NILs with the peak expression at 12 HAI in Apogee73S2 and at 24 HAI in Apogee (Figure 5C). However, *TaNPR2* was not differentially expressed between the NILs at all the time points tested (Figure 5D). *TaNPR3* also showed significantly higher expression in Apogee73S2 between 12 and 48 HAI (Figure 5E). Taken together, both *TaNPR1* and *TaNPR3* may play important roles in enhancing wheat FHB resistance through SA signal transduction.

### Functional Evaluation of Salicylic Acid-Related Key Genes Using *Barley Stripe Mosaic Virus*-Mediated Gene Silencing

**FIGURE 6:**
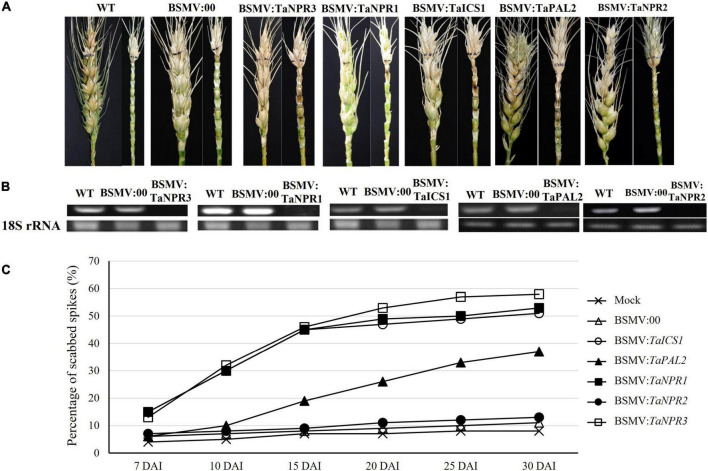
Effects of the five genes in SA pathways and signaling that were silenced by *barley stripe mosaic virus*-mediated gene silencing (BSMV-VIGS) from Sumai 3 on FHB resistance. **(A)** FHB symptoms on wheat spikes with BSMV-VIGS silenced genes. **(B)** Gene expression profiles for the five VIGS-treated genes in wheat spikes were evaluated by semi-quantitative RT-PCR. The wheat *18S rRNA* gene was used for endogenous reference for normalization. Each treatment had three biological replicates, and each biological replicate had three technical replicates. **(C)** The percentage of scabbed spikelets in the spikes with and without VIGS-silenced genes from Sumai 3 at 7, 10, 15, 20, 25, and 30 days after inoculation (DAI). BSMV:00, BSMV with blank vector.

Transient gene silencing was successfully used to silence the two genes on the SA synthesis pathway and three genes on SA signaling pathway. The Sumai 3 plants with silenced *TaICS1*, *TaNPR1*, or *TaNPR3* showed significantly higher (*p* < 0.05) FHB severity (∼45%) than those for wild-type and non-silenced controls (10%) at 15 DAI (Figure 6). The plants with silenced *TaPAL2* gene had no significant difference in PSS compared to the Sumai 3 controls until 10 DAI, and then the PSS in the *TaPAL2-*silenced plants was significantly increased (*p* < 0.05) after 15 DAI and reached 38% at 30 DAI. However, PSS has not changed even 30 DAI in the plants carrying silenced *TaNPR2*. Those results confirmed that *TaICS1* in the SA synthesis pathway and *TaNPR1* and *TaNPR3* in the SA signaling pathway play important roles in wheat FHB resistance.

## Discussion

### Black Necrotic Lesion Restrained *F. graminearum* Spread in Wheat

The typical early visible FHB symptoms include brownish water-soaked spots on the glume of an inoculated spikelet. With the infection progression, all the infected spikelets may dry up with an appearance similar to ripen spikelets and die quickly. These bleached, premature spikelets due to FHB damage usually mix with green spikelets in a spike in the later grain filling stage in moderately resistant or susceptible cultivars, but the apical part or entire wheat spike may dry up completely as the fungal infection progresses through rachis that cut off the water and nutrient supplies to these apical spikelets in highly susceptible genotypes (Kang and Buchenauer, 2000; Bai et al., 2002). However, in highly resistant genotypes such as Sumai 3, only small BNLs were observed on the initially infected glumes and the majority of the spike remains green till maturity as seen in this study (Figure 1 and Supplementary Figure 1). Holmes (1929) noticed similar local lesions that were induced by TMV. The local lesion has been considered a resistance mechanism that limited the spread of the virus from the initial inoculation point (Loebenstein, 2009). In this study, we observed only a few hyphae around the BNL induced by FHB infection (Figure 1) and showed that the BNL was closely associated with ROS burst (Supplementary Figure 3), SA pathway, and flavonoids metabolites. The BNLs appeared to be HR-like symptoms that were induced by *F*. *graminearum*, especially in the FHB-resistant wheat genotypes, and it significantly enhanced the FHB resistance by restraining the growth and spread of the pathogen within wheat tissue. The integrated metabolomic and transcriptomic analyses of the inoculated samples in this study suggested that the upregulated DEGs induced by the two low virulent isolates (R40 and R64) mainly encoded proteins for phytohormone signaling pathways and for the redox state, secondary metabolites, cell wall, abiotic stress pathways, and phenolamine and flavonoids metabolic pathways to form BNLs that restricted *F. graminearum* extension (Figure 2).

### Effect of Salicylic Acid on Wheat Fusarium Head Blight Resistance

Salicylic acid (SA) is a small phenolic compound that regulates plant growth and development (Rivas-San Vicente and Plasencia, 2011); it also serves as a critical signal to activate the expression of disease resistance genes (Delaney et al., 1994; An and Mou, 2011). The SA biosynthesis in *Arabidopsis* is primarily *via* an isochorismate synthase (ICS) pathway. A distinct pathway utilizing phenylalanine ammonia-lyase (PAL) as the substrate may also contribute to SA accumulation (Wildermuth et al., 2001; Dempsey et al., 2011). However, the SA biosynthetic pathway remains largely unknown in *Triticeae*. Hao et al. (2018) cloned an *ICS* gene and seven *PAL* genes from barley (*Hordeum vulgare*) and studied their functions by overexpression and suppression of these genes in barley, and they found that suppression of *ICS* reduced plant FHB resistance, but suppression of *PAL* expression did not. Suppression of *ICS* might result in a reduction in SA accumulation that weakens defense responses during pathogen infection; therefore, they concluded that *ICS* likely played a unique role in barley SA biosynthesis (Hao et al., 2018). In the current study, *TaICS1* and *TaPAL2*, the two key genes for SA synthesis, in resistant cultivar Sumai 3 were transiently silenced by VIGS, and only the *TaICS1* silenced plants significantly increased PSS compared to its wide type genotype at 15 DAI, suggesting that *TaICS1* might play an important role in wheat FHB resistance by regulating SA synthesis pathway in wheat (Figures 5, 6). Interestingly, DEG in SA biosynthesis pathway was not detected in the RNA-seq study where the tissue was collected at 4 DAI, which might be due to the observation that these genes in the SA biosynthesis pathway have been activated at earlier stages of FHB infection. This assumption was supported by the observation that SA content in the Sumai 3’s spike tissue increased quickly after inoculation and reached the peak at 12 HAI, which is highly consistent with the gene expression pattern of *TaICS1* (Figure 5). Furthermore, we confirm that the *TaICS1* gene plays a major role in wheat SA biosynthesis and high SA content in the early infection stage was highly related to reactive oxygen burst, BNL formation, and FHB resistance (Supplementary Figure 4).

In *Arabidopsis*, a non-expression of a pathogenesis-related (PR) gene (*AtNPR1*) is considered a key regulator of SAR, which regulates *PR* gene expression through interaction with the TGACG motif-binding factor family of transcription factors (Zhang et al., 1999; Zhou et al., 2000). Mutants of *AtNPR1* did not respond to various SAR-inducing treatments, showed a low expression of the PR genes, and exhibited higher disease susceptibility (Cao et al., 1997; Dong, 2004; Makandar et al., 2006). In wheat, overexpression of *AtNPR1* and *Secale cereale*-*NPR1* in an FHB-susceptible wheat cultivar enhanced wheat FHB resistance (Makandar et al., 2006; Yu et al., 2017). However, Gao et al. (2013) reported that the transgenic wheat lines expressing *AtNPR1* were resistant to *Fusarium* in spikes, but highly susceptible at the seedling stage, suggesting that NPR1 may have dual functions in regulating defense responses in plants. In the current study, silencing *TaNPR2*, an *AtNPR1* homolog in wheat, in Sumai 3 did not significantly change FHB resistance, but silencing *TaNPR1* and *TaNPR3* (two *TaNPR1* homologs) in Sumai 3 significantly reduced wheat FHB resistance (Figure 6), suggesting different *NPR* functions between wheat and *A*. *thaliana*. *TaNPR1* and *TaNPR3* genes likely play an important role in SA signal transduction to facilitate wheat responses to FHB infection.

### The Effects of Salicylic Acid and Jasmonic Acid Interaction on Wheat Fusarium Head Blight Resistance

Jasmonic acid and SA are two key endogenous phytohormonal signal molecules that regulate plant resistance to diverse pathogens (Makandar et al., 2012; Yang et al., 2015). SA primarily regulates defense mechanisms against biotrophic and hemibiotrophic pathogens, whereas JA primarily contributes to resistance against necrotrophic pathogens, which have been well characterized in *A. thaliana* (Glazebrook, 2005; Koornneef and Pieterse, 2008). For resistance to *F. graminearum*, both SA and JA may be required for the basal resistance response in *Arabidopsis* (Makandar et al., 2010). However, JA signaling shows dichotomous functions in both *Arabidopsis*-*F*. *graminearum* and wheat-*F*. *graminearum* interactions, in which JA attenuated SA signaling during the early infection stages and promoted defense against *F. graminearum* during the later infection stages (Makandar et al., 2010). JA and its methyl ester, methyl jasmonate (MeJA), and plant lipid derivatives may play a role in plant responses to wound and pathogen attacks. In the current study, the application of exogenous SA and MeJA to detached wheat leaves and spikes induced local or systemic responses and variations in FHB severity in the treated plants. MeJA reduced wheat FHB resistance and inhibited the BNL formation on the detached leaves due to attenuation of SA signaling in resistant wheat Sumai 3 (Supplementary Figure 2). Therefore, the SA signaling pathway more likely contributes to the BNL formation.

Furthermore, the detached leaves showed similar responses to early-stage infection by *F*. *graminearum* and can be used as alternative materials to study the spread of *F*. *graminearum* in wheat tissue. The current study showed that the effect of interaction between SA and JA on wheat FHB resistance as measured by FIR, DR, and PSS per spike is time-dependent. SA defense against *F*. *graminearum* infection started in an early stage (within 24 HAI) by inducing a local resistant response; however, the resistance disappeared if SA was applied at a later infection stage (after 48 HAI), probably due to the reactive oxygen burst (Makandar et al., 2012; Sorahinobar et al., 2016). Although the early stage MeJA treatments facilitated the spread of *F*. *graminearum* to rachises, the application of exogenous MeJA effectively limited the late spread of *F*. *graminearum* within a spike, resulting in low final PSS in all-time points of MeJA treatments (Figure 4I).

### Fusarium Head Blight Resistance-Related Metabolites in Wheat

Hamzehzarghani et al. (2005) analyzed the metabolic profiles in spikelets of wheat cultivars, Roblin and Sumai 3, using GC/MS and developed a method to discriminate FHB resistance levels using metabolic profiling (Hamzehzarghani et al., 2005). They found several highly abundant fatty acids, aromatic compounds, p- and m-coumaric acids, myo-inositols and other sugars, and malonic acids in resistant cultivar Sumai 3, but only amino acids, fatty acids, and aromatics in the susceptible cultivar Roblin (Hamzehzarghani et al., 2005). In barley, metabolomics was used to identify the metabolites that are related to FHB resistance. The resistance-related metabolites mainly include phenylpropanoid, flavonoid, fatty acid, and terpenoid metabolic pathways (Bollina et al., 2010). Recently, an integrated analysis of metabolomics and transcriptomics of infected wheat plants identified 789 differentially accumulated metabolites, including flavonoids, phenolamides, tryptamine derivatives, and phytohormones, and revealed altered expression of more than 100 genes that functioned in the biosynthesis or regulation of these pathways (Su et al., 2020). In the current study, a large number of amino acid derivatives, flavones, nucleotide, and its derivatives, and quinate derivatives were detected in both low and high virulent isolates inoculated samples, thus they were most probably induced by *F*. *graminearum* infection, which is consistent with the previous reports (Hamzehzarghani et al., 2005). Similarly, a large number of flavonoid and terpenoid metabolites were identified from the samples that were inoculated with low virulent *F*. *graminearum* isolates, which is consistent with Su et al. (2020). Furthermore, flavonoids are widely present in plants and belong to a biologically important and chemically diverse group of secondary metabolites with diverse subgroups (Treutter, 2005, 2006). In the phenolic phytoalexin subgroup, the isoflavonoids, phenylpropanoids, and simple phenolics have been well characterized. In this study, exogenous application of apigenin and neohesperidin on the detached leaves of all selected wheat cultivars reduced *Fusarium* infection and induced BNL surrounding the infection sites; application of proanthocyanidins and spermidine, however, did not improve FHB resistance. Integrated metabolomic and transcriptomic analyses suggest that the flavonoid metabolic pathway might function differently between FHB resistant and susceptible wheat cultivars, and play an important role in the formation of BNLs in resistant genotypes in response to *F*. *graminearum* infection (Figure 2). Although direct evidence for SA to increase the accumulation of flavonoids was not detected in this study, exogenous SA has been known to boost the accumulation of flavonoids in several plant species (Xu et al., 2009; Tounekti et al., 2013). Moreover, SA has been reported to trigger the expression of some core flavonoid biosynthetic genes and lead to the accumulation of flavonoid phytoalexins (Ahuja et al., 2012). In this study, all the five DEGs induced by the low virulent *F*. *graminearum* isolates were upregulated and all the eight DEGs induced by the high virulent *F*. *graminearum* isolates were downregulated in the flavonoids metabolic pathway (Figure 2), which support roles of flavonoids metabolic pathway in FHB resistance. Further research is needed to determine the effects of SA on the biosynthesis of particular flavonoids, and to establish the relationship between the flavonoids’ biosynthetic pathways and specific individual flavonoids leading to FHB resistance.

## Data Availability Statement

The datasets presented in this study can be found in online repositories. The names of the repository/repositories and accession number(s) can be found below: https://www.ncbi.nlm.nih.gov/search/all/?term=PRJNA842823. The accession number of the raw data is PRJNA842823.

## Ethics Statement

The authors declare that the experiments comply with the current laws of the country in which they were performed.

## Author Contributions

LZ, PS, BH, HWW, and LK designed the experiments. LZ, PS, BH, YF, HYW, WL, JZ, WG, and SX performed the experiments. XM, SW, and AL contributed to plant materials. LZ, PS, and GB wrote the manuscript. All authors have read and approved the manuscript.

## Conflict of Interest

The authors declare that the research was conducted in the absence of any commercial or financial relationships that could be construed as a potential conflict of interest.

## Publisher’s Note

All claims expressed in this article are solely those of the authors and do not necessarily represent those of their affiliated organizations, or those of the publisher, the editors and the reviewers. Any product that may be evaluated in this article, or claim that may be made by its manufacturer, is not guaranteed or endorsed by the publisher.

## Footnotes

1
https://mapman.gabipd.org/mapman

2
http://mapman.gabipd.org/web/guest/Mercator

3
www.shimadzu.com.cn/

4
www.appliedbiosystems.com.cn/
