# Supplementary material for: The Black Necrotic Lesion Enhanced Fusarium graminearum Resistance in Wheat
Source: Front Plant Sci. 2022 Jun 30;13:926621. doi: 10.3389/fpls.2022.926621 (PMC9280303; doi:10.3389/fpls.2022.926621)
Supplement: Supplementary file 2 [file Presentation_1.PPTX]

## Slide 1
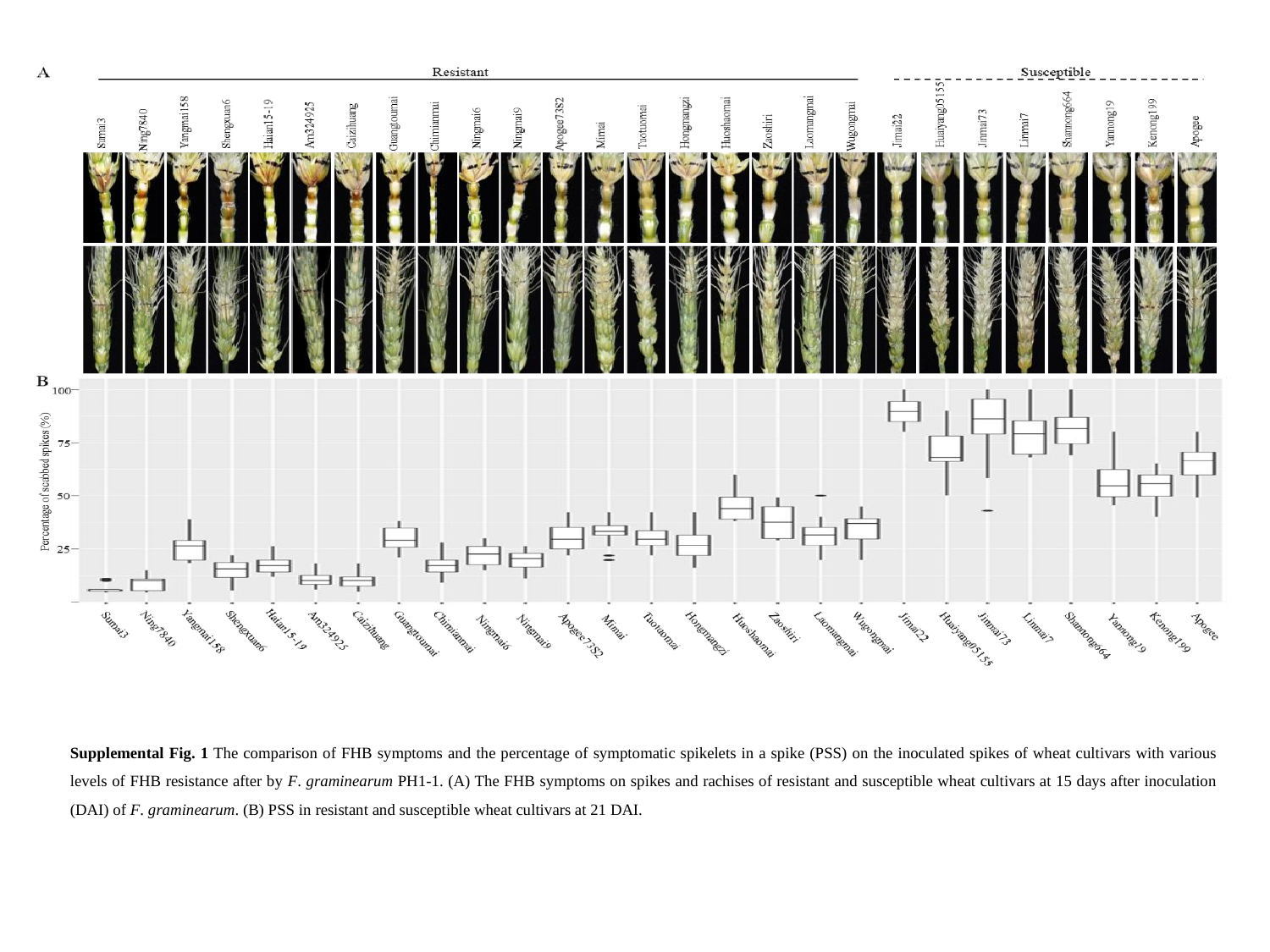

Supplemental Fig. 1 The comparison of FHB symptoms and the percentage of symptomatic spikelets in a spike (PSS) on the inoculated spikes of wheat cultivars with various levels of FHB resistance after by F. graminearum PH1-1. (A) The FHB symptoms on spikes and rachises of resistant and susceptible wheat cultivars at 15 days after inoculation (DAI) of F. graminearum. (B) PSS in resistant and susceptible wheat cultivars at 21 DAI.

## Slide 2
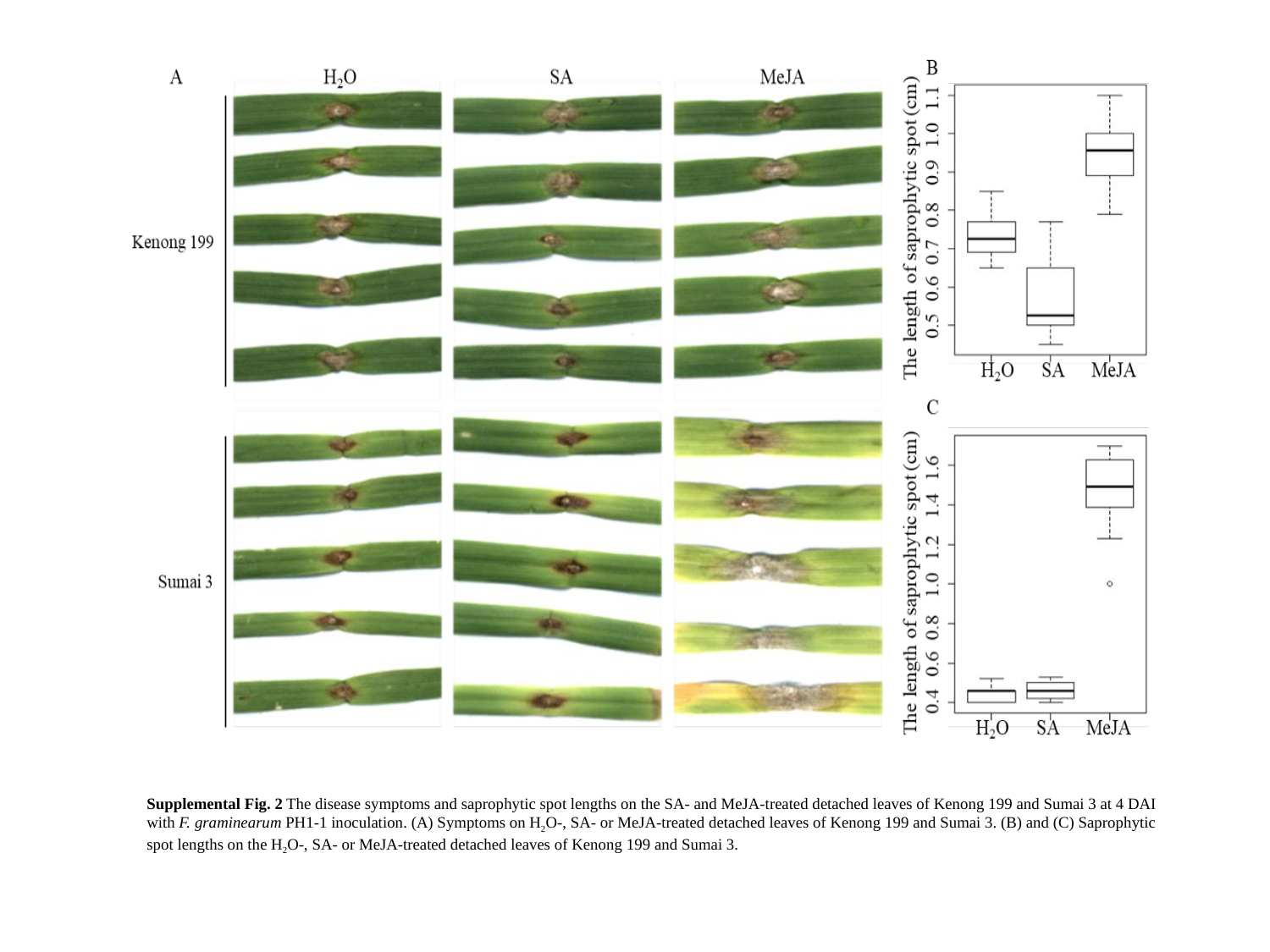

Supplemental Fig. 2 The disease symptoms and saprophytic spot lengths on the SA- and MeJA-treated detached leaves of Kenong 199 and Sumai 3 at 4 DAI with F. graminearum PH1-1 inoculation. (A) Symptoms on H2O-, SA- or MeJA-treated detached leaves of Kenong 199 and Sumai 3. (B) and (C) Saprophytic spot lengths on the H2O-, SA- or MeJA-treated detached leaves of Kenong 199 and Sumai 3.

## Slide 3
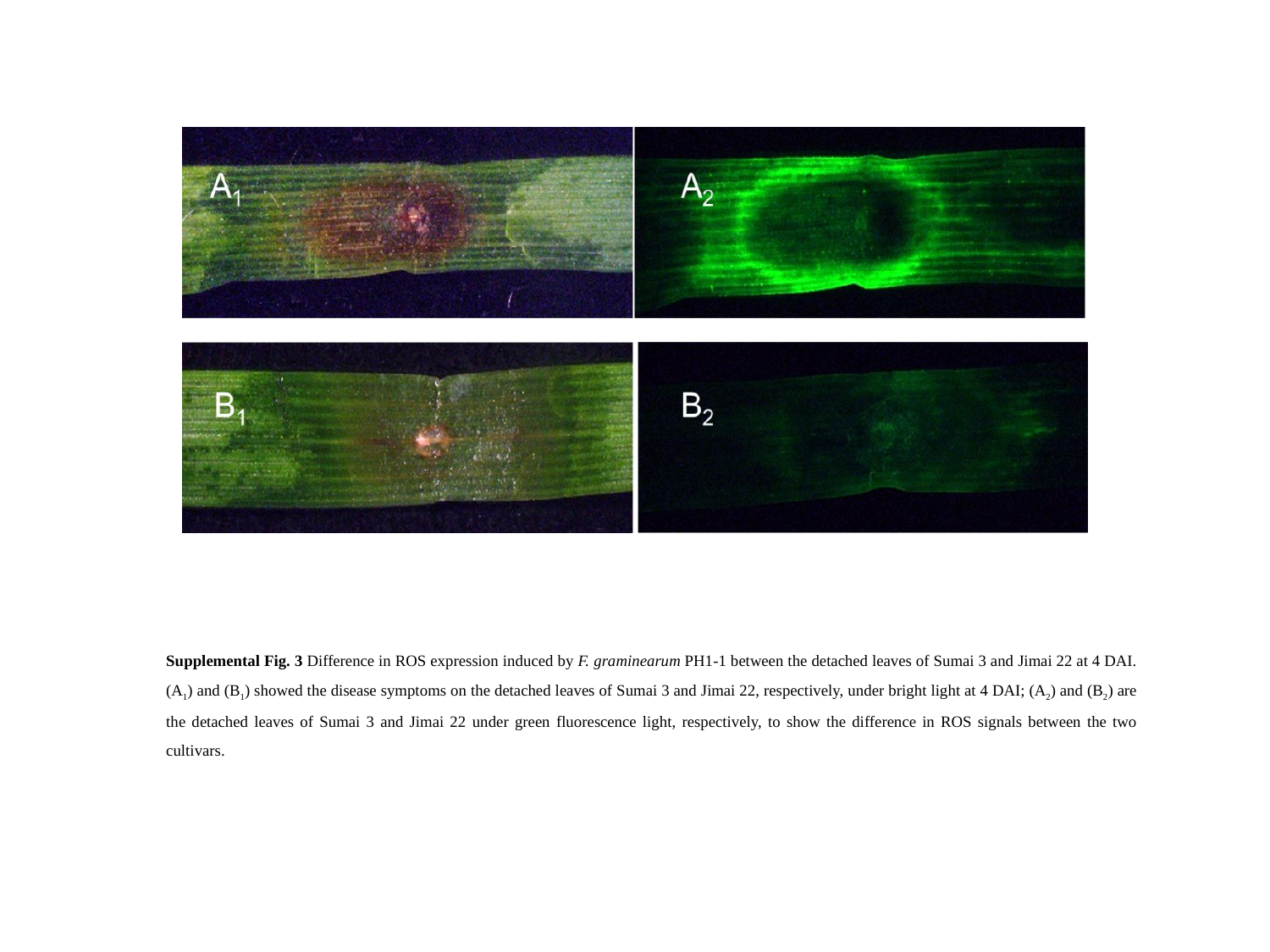

Supplemental Fig. 3 Difference in ROS expression induced by F. graminearum PH1-1 between the detached leaves of Sumai 3 and Jimai 22 at 4 DAI. (A1) and (B1) showed the disease symptoms on the detached leaves of Sumai 3 and Jimai 22, respectively, under bright light at 4 DAI; (A2) and (B2) are the detached leaves of Sumai 3 and Jimai 22 under green fluorescence light, respectively, to show the difference in ROS signals between the two cultivars.

## Slide 4
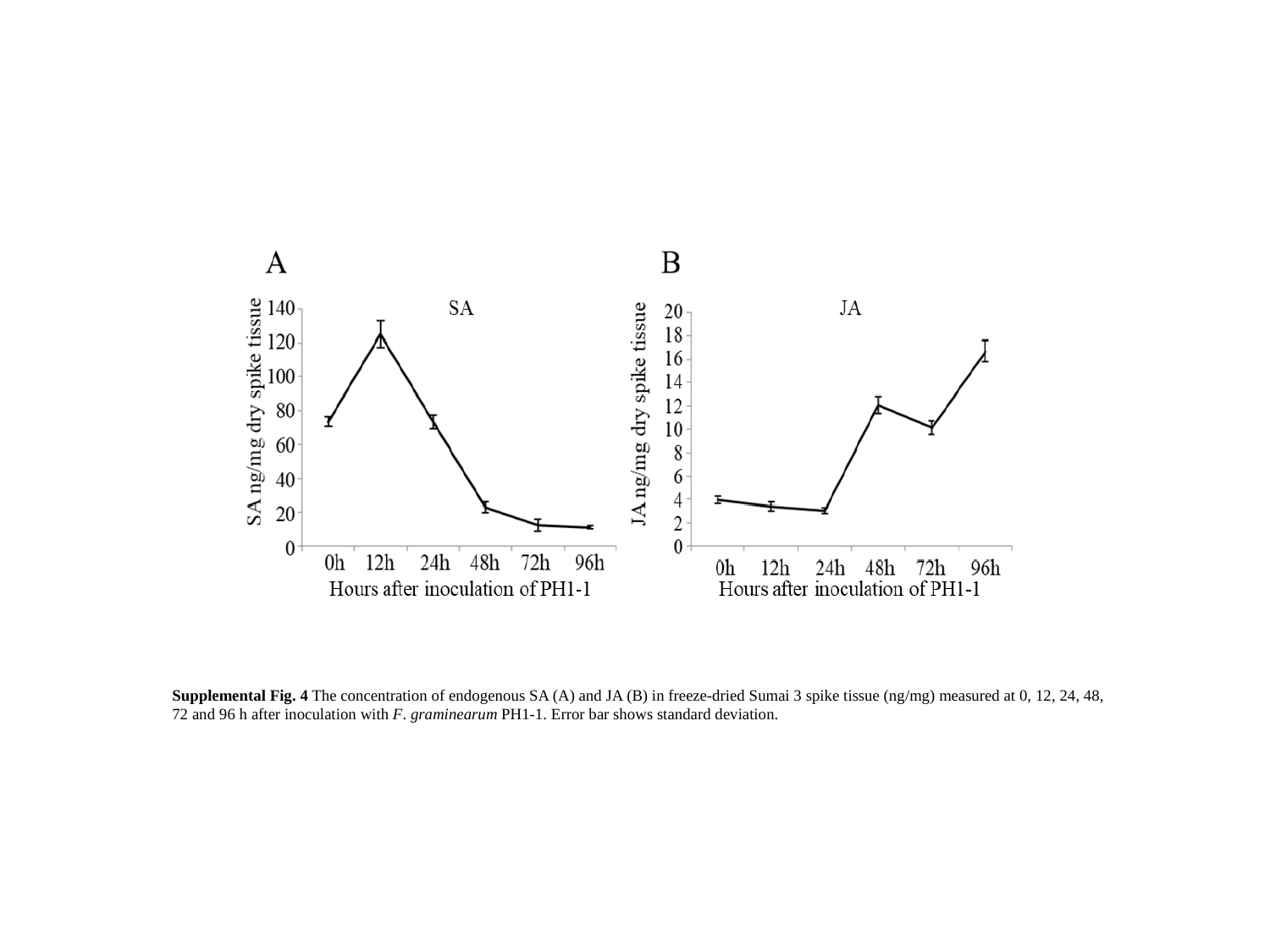

Supplemental Fig. 4 The concentration of endogenous SA (A) and JA (B) in freeze-dried Sumai 3 spike tissue (ng/mg) measured at 0, 12, 24, 48, 72 and 96 h after inoculation with F. graminearum PH1-1. Error bar shows standard deviation.
